# Synthesis of Lanthanum-Modified Natural Magnetite: Characterization and Valorization for Phosphorus Recovery from Aqueous Solutions

**DOI:** 10.3390/ma18102283

**Published:** 2025-05-14

**Authors:** Hamed Al-Nadabi, Salah Jellali, Wissem Hamdi, Afrah Al-Tamimi, Ahmed Al-Raeesi, Ahmed Al-Sidairi, Waleed Al-Busaidi, Ahlam Al-Hanai, Khalifa Al-Zeidi, Malik Al-Wardy, Mejdi Jeguirim

**Affiliations:** 1 Centre for Environmental Studies and Research, Sultan Qaboos University, Al-Khoud 123, Muscat P.O. Box 17, Oman; hamed@squ.edu.om (H.A.-N.); aalraeesi@squ.edu.om (A.A.-R.); sidairi@squ.edu.om (A.A.-S.); alzeidi@squ.edu.om (K.A.-Z.); mwardy@squ.edu.om (M.A.-W.); 2 Higher Institute of the Sciences and Techniques of Waters, University of Gabes, Gabes 6033, Tunisia; wissemhemdi@yahoo.fr; 3 College of Agricultural and Marine Sciences, Sultan Qaboos University, Al-Khoud 123, Muscat P.O. Box 17, Oman; waleedm@squ.edu.om; 4 The Institute of Materials Science of Mulhouse (IS2M), UMR 7361, University of Haute Alsace, CNRS, P.O. Box 2488, 68100 Mulhouse, France; mejdi.jeguirim@uha.fr

**Keywords:** magnetite deposits, chemical modification, nutrients, adsorption characteristics, efficiency

## Abstract

In this research work, a natural sample from an Omani magnetite (MG) deposit was used for the synthesis of a magnetite decorated with ferrihydrite (MG-Fh), and two lanthanum (La)-modified materials at mass percentages of 5% (MG-Fh-La-5) and 15% (MG-Fh-La-15). These materials were first characterized using various analytical techniques. Then, their phosphorus (P) recovery efficacy from aqueous solutions was studied in batch mode under a wide range of experimental conditions. The characterization results show that compared to the raw feedstock, MG-Fh, MG-Fh-La-5, and especially MG-Fh-La-15 have improved structural, textural, and surface chemistry properties. Adsorption tests indicate that due to the deposition of high contents of lanthanum oxides on its surface, the MG-La-15 exhibited a large P uptake capacity (34.5 mg g^−1^), which is significantly superior to those determined for MG-La-5 (24.3 mg g^−1^), MG-Fh (12.4 mg g^−1^), and various engineered materials published in the literature. Moreover, these materials retain an interesting ability to recover P from real wastewater with a highest adsorbed mass of 27.3 mg g^−1^, observed for MG-La-15. The P recovery seems to involve both physical and chemical mechanisms, including electrostatic interactions and complexation. This research work shows that La-modified magnetite can be considered a promising and eco-friendly material for P recovery from liquid effluents.

## 1. Introduction

Huge amounts of phosphorus (P) are globally discharged in wastewater [1]. This quantity was evaluated to be around 1.5 million tones (MT) in 2010 and will reach 1.6 to 2.4 MT by 2050 [2]. Along with nitrogen, P is regarded as a serious threat to the quality and biodiversity of surface water due to the eutrophication process [3]. The restoration of eutrophic water bodies is usually a long and costly task [4]. At the same time, P is an essential element for agriculture. According to International Fertilizer Association (IFA: https://www.ifastat.org/ (accessed on 14 April 2025)), in 2023, the worldwide production of P from natural deposits was evaluated to be 23.1 MT. The longevity of these finite natural reserves is controversial, since the predicted duration until their complete exhaustion was found to vary from centuries to only a few decades [5]. Therefore, P recovery from wastewater for further subsequent use in agriculture as a biofertilizer can reduce the exploitation pressure on P reserves and at the same time, significantly decrease the surface water quality deterioration risk through eutrophication. Numerous methods have been used for P recovery from wastewater. They mainly include struvite crystallization (either as MgNH_4_PO_4_x6H_2_O or MgKPO_4_x6H_2_O), P accumulation into specific micro-organisms, and adsorption onto natural or modified materials [6,7]. Unlike the former technologies that require stringent experimental conditions, adsorption is usually recommended due to its simplicity, practicality, low cost, and eco-friendliness [8,9].

Various raw and modified natural materials have been used for P recovery from wastewater. They mainly encompass feedstock from lignocellulosic biomass and sludge [8,9], animal manure [10], zeolite [11], and synthetic/natural magnetite [12]. Natural magnetite has the advantages of abundance, eco-friendliness, and also low cost. However, in its raw form, it has poor to moderate physico-chemical properties, which usually limit its capacity in recovering P to less than 5 mg g^−1^ [12,13]. For this reason, different modification methods have been used for the improvement of magnetite properties, and consequently, the enhancement of their ability in recovering P from aqueous solutions. In this context, magnetite was modified with lanthanum (La) [14], La and Zirconium (Zr) [15], zinc (Zn) [16], and a mixed solution of magnesium and iron (Mg/Fe) [17,18]. These magnetite-modified materials exhibited better properties and higher P recovery capacities in comparison with the raw feedstock. For instance, relatively high P recovery capacities of 253.8, 98.8, and 49.1 mg g^−1^ were reported for Fe_3_O_4_/La(OH)_3_ nanocomposite [12], Mg/Fe-modified magnetite [17], and La-Zr-modified magnetite [15], respectively. However, these studies were carried out using synthetic magnetite through the co-precipitation method of Fe(III)/Fe(II) mixed solution at a high alkaline pH (more than 10) [19]. The latter method presents shortcomings of a high cost, complex preparation conditions, less stability, and environmental pollution risk [20].

Ferrihydrite (Fe_10_O_14_(OH)_2_) is an iron oxyhydroxide nanomaterial that can be synthesized from Fe(III) solution and adjusted to a pH value of around 7 [21,22,23]. Compared to raw magnetite, this synthetic Fh-decorated magnetite was found to be more efficient in recovering P [21] and also in removing heavy metals from aqueous solutions, such as antimony (Sb(III) and Sb(V)) [24]. However, to reduce the preparation cost, ferrihydrite may be synthesized from natural magnetite through its agitation in acidic solutions, which releases Fe(III) and can be later transformed into nano-ferrihydrite by adjusting this solution’s pH to around 7 [14]. The resulting ferrihydrite-coated magnetite typically has a high surface area and is rich in hydroxyl groups, which can contribute to the efficient adsorption of pollutants [22]. This capacity can be further improved if this ferrihydrite-coated magnetite is modified with lanthanum for nutrient recovery [25], and with other chemicals (i.e., phosphates) for heavy metal removal [26] from aqueous solutions.

The use of La for the decoration of Ferrihydrite-coated magnetite has the following main advantages: (i) La is a relatively inexpensive rare earth element, (ii) La is an environmentally benign compound, (iii) La nanoparticles are electron pair acceptors and should efficiently adsorb oxyanions in general and P, in particular, and (iv) this magnetic product can be easily separated from aqueous solutions through magnets. Few studies have synthesized La-modified ferrihydrite-coated magnetite [25]. However, the effect of the percentage of La-modified ferrihydrite-coated magnetite on the properties of the synthesized materials, as well as their efficiency in recovering P under a wide range of experimental adsorption conditions (i.e., contact time, aqueous pH, and material dose, etc.), has not yet been precisely assessed. Moreover, the impact of using actual wastewater instead of synthetic solutions on P recovery efficiency is usually ignored when dealing with La-modified materials [8]. In the present work, we intend to fill in these research gaps by using lanthanum-modified natural magnetite decorated with ferrihydrite at mass ratios (La:MG) of 5% and 15%. Practically, the current research investigation has the main following purposes: (i) the synthesis of lanthanum-modified natural magnetite (from Oman) at mass ratios of 0%, 5%, and 15%, (ii) the characterization of these synthesized materials using different analytical techniques, (iii) an investigation of these adsorbents’ performance in recovering P from liquid effluents under various experimental conditions including contact time, the aqueous solutions’ initial pH, the adsorbents’ dose, the presence of competitive ions, the initial P concentrations, and the use of actual wastewater and, (iv) an exploration of the main possibly involved mechanisms in the overall P recovery process.

## 2. Materials and Methods

### 2.1. Magnetite Preparation

The used natural magnetite was collected from Al-Nabaa mountain in Al-Qabel city, Oman. Firstly, this material was thoroughly washed with distilled water to remove impurities. Then, it was dried at 85 °C for 24 h. After that, this dried material was ground using a mechanical grinder. The fraction with dimensions lower than 300 μm was stored in a glass container and used for the preparation of the La-modified magnetite.

### 2.2. Synthesis of Lanthanum-Modified Magnetite Decorated with Ferrihydrite

**Figure 1:**
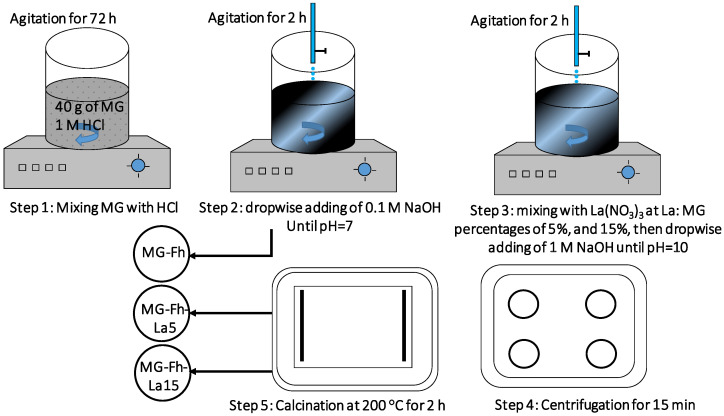
Schematic representation of the used protocol for the synthesis of the La-modified materials.

The synthesis of magnetite decorated with ferrihydrite was carried out as follows (Figure 1): Firstly, 40 g of raw magnetite was agitated for 72 h in 1 L of 1 M hydrochloric acid (HCl) solution using a magnetic stirrer (Cole Parmer SHP-200-s, Chicago, IL, USA) at a speed of 200 rpm. Then, 0.1 M of NaOH solution was added dropwise until reaching a pH of 7.0. Afterwards, this solution was stirred for 2 h. The obtained material was labeled MG-Fh.

The following steps were used for the synthesis of La-modified MG-Fh: At the beginning, specific masses of La(NO_3_)_3_x6H_2_O were added to the MG-Fh suspensions to obtain La/magnetite mass ratios of 5% and 15%. Then, 1 M NaOH solution was added dropwise to this suspension until reaching a pH of 10 and then agitated for 2 h. Afterwards, this solution was centrifuged at 3000 rpm for 15 min, and the separated solid matrixes were rinsed three times with distilled water and then calcined at 200 °C in a laboratory furnace for 2 h. The resulting solids were stored in glass flasks for ulterior use for characterization and adsorption studies. They were labeled MG-Fh, MG-Fh-La-5, and MG-Fh-La-15, corresponding to La/MAG mass ratios of 0%, 5%, and 15%, respectively.

### 2.3. Materials Physico-Chemical Characterization

The four used materials (MAG, MG-Fh, MG-Fh-La-5, and MG-Fh-La-15) were characterized using several analytical techniques. This characterization step involves the assessment of the following: (i) The surface morphology and qualitative elemental composition via a scanning electron microscope (SEM) coupled with energy dispersive X-Ray (EDS) (JSM-7800F, Jeol, Tokyo, Japan). (ii) The elemental composition through the non-destructive X-ray fluorescence (XRF) method (NEX QC, Rigaku Tokyo, Japan). (iii) The crystallographic structure via X-ray diffraction (XRD) (MINFLEX 600, Rigaku, Tokyo, Japan), for diffraction angles (2θ) varying between 10° and 80°. The obtained diffractogram interpretation and the contained phase identification were assessed using the Panalytical X’Pert HighScore software, v5.1b and the database established by the international center for diffraction data (ICDD). (iv) The textural properties through the exploitation of nitrogen adsorption/desorption isotherms at 77 K to determine the BET surface areas and total pore volumes (TPVs) using a Micrometrics instrument (ASAP-2020, Ottawa, ON, Canada). (v) The functional groups’ composition using a Fourier Transform Infrared (FTIR) apparatus (ALPHA II, Bruker, Leiderdorp, The Netherlands). For each sample, an adsorbent material to KBr mass ratio of 2/100 was ground and pressed into 1 cm diameter disk with 3.5 tons of pressure. This sample was then analyzed using the FTIR device at a spectral resolution of 1 cm^−1^, for wavenumbers between 400 and 4000 cm^−1^. (vi) The pH at the point of zero charge (pHpzc) of the materials’ surface using the pH drift method [27]. This method consists of agitating 0.2 g of the materials in 50 mL of 0.1 M NaCl solutions at initial pH values (pHi) varying between 3 and 11. The pHpzc values were determined from the graphs giving the difference between the final pH (pHf) and pHi (∆pH = pHf − pHi) versus pHi. They correspond to the intersection of this curve with the abscise axis (pHi).

### 2.4. Batch Phosphorus Recovery

#### 2.4.1. Phosphorus Solutions’ Preparation and Analysis

Disodium hydrogen phosphate (Na_2_HPO_4_x6H_2_O) was purchased from Sigma-Aldrich and used for the preparation of a stock P solution at a concentration of 3000 mg P L^−1^. This solution was used for the preparation of the desired P concentrations through dilution with distilled water. In addition, solutions of 0.1 M NaOH and HCl (acquired from Sigma-Aldrich, St. Louis, MO, USA) were employed for the adjustment of the initial pH values of these prepared samples. The pH values were measured using a bench pH meter (Mettler Toledo, OH, USA).

The P concentrations in the aqueous samples were assessed using the Fleury method. The intensity of the obtained yellow color was measured using a UV-visible spectrometer (UV-1900i, Shimadzu; Kyoto, Japan) at a wavelength of 430 nm.

#### 2.4.2. Phosphorus Adsorption Protocol and Data Analysis

The P recovery efficiency from aqueous solutions by the three materials (MG-Fh, MG-Fh-La-5, and MG-Fh-La-15) was carried out in batch mode with synthetic solutions. It consisted of agitating a mass of a given adsorbent in 50 mL of a solution at a fixed P concentration using a multi-position magnetic stirrer (Gallenkamp, Cambridge, UK) at a shaking speed of 600 rpm. To obtain a better understanding of the P recovery characteristics, diverse adsorption experiments were conducted to determine the effect (i) the contact times for values varying between 1 min and 24 h, (ii) the initial aqueous pH (from 3 to 11), (iii) the adsorbent dose from 0.5 to 50 g L^−1^, (iv) the presence of anions such as chlorides, sulfates, nitrates, and carbonates, and (v) the P initial concentrations (in the range between 5 and 100 mg L^−1^). During these assays, only one parameter was varied, and all others were kept constant and equal to the default values. These default values were fixed to 24 h for the contact time, 6.2 for the initial aqueous pH (not adjusted), 66.0 mg L^−1^ for the P concentration, 1 g L^−1^ for the adsorbent dose, and 25 °C for the temperature. The variation range as well as the default values of these factors were chosen based on preliminary investigations and former comparable published studies [28,29].

At the end of the agitation period, liquid samples (before and after P adsorption) were filtrated through 0.22 μm polyvinylidene difluoride (PVDF) filters (Whatman, Maidstone, UK) and then analyzed via a UV-visible spectrometer, as mentioned in the section above. At a given time ‘t’, the P adsorption efficiency is judged based on both the adsorbed amount (q_t_ (mg g^−1^) and the corresponding removal yield (R_t_ (%)). These two parameters are calculated as below:

(1) $$q_{t}=\frac{\left(C_{0}-C_{t}\right)*V}{m}$$

(2) $$R_{t}(\%)=\frac{\left(C_{0}-Ct\right)}{C_{0}}\times 100$$

where C_0_ and C_t_ (mg L^−1^) are the P concentrations at the beginning and after a contact time ‘t’ of the adsorption assay, respectively. V and m are the volume of the liquid sample (L) and the mass of the magnetite-based adsorbent (g), respectively.

Moreover, to obtain a better understanding of the main mechanisms involved during the P recovery process, the obtained kinetic data were fitted to three well-known models, specifically the pseudo-first-order (PFO), pseudo-second-order (PSO), and diffusion models (DM). Furthermore, the isotherm experimental data were fitted with three famous models, namely the Freundlich, Langmuir, and Dubinin–Radushkevich (D-R) models. Besides acquiring insights into the P recovery mechanisms, this isotherm modeling step allows for the determination of the materials’ adsorption capacities. Table S1 gives details about these models and the related parameters.

The goodness-of-fit between the kinetic/isotherm experimental and calculated curves was evaluated through the determination of their correlation coefficients (R^2^) and also the mean absolute percentage deviation between the measured and calculated kinetic (MAPD_K_) and isotherm (MAPD_I_) data, as follows:

(3) $$MAPD_{K}\;=\;\frac{\sum \left|\frac{q_{t,meas}\;-\;q_{t,calc}}{q_{t,meas}}\;\right|}{N}\;*\;100$$

(4) $$MAPD_{I}\;=\;\frac{\sum \left|\frac{q_{e,\exp}\;-\;q_{e,calc}}{q_{e,meas}}\;\right|}{N}\;*100$$

where q_t,meas_ and q_t,calc_, and q_e,meas_ and q_e,calc_ are the measured and calculated amounts of recovered P at time ‘t’ and at equilibrium, respectively. N denotes the number of experimental sets.

Batch assays were conducted in triplicate and the corresponding average values are given in the enclosed plots.

#### 2.4.3. Phosphorus Recovery from Actual Wastewater

The efficiency of the tested magnetite-based adsorbents in recovering P from actual wastewater was assessed under the following conditions: a contact time of 24 h, a dose of 1 g L^−1^, at a natural pH (without adjustment), and at a doped P concentration of 66.0 mg L^−1^. The corresponding results were compared to those obtained for synthetic solutions under the same conditions to assess the impact of wastewater composition and complexity. The actual wastewater was sampled from an urban wastewater treatment plant located in Muscat, Oman.

#### 2.4.4. Adsorbent Regeneration

The P-loaded adsorbents’ regeneration was carried out for 4 consecutive adsorption/desorption cycles. For each given cycle, the P adsorption was carried out for a contact time of 24 h, at a natural pH, and at a dose of 20 g L^−1^ (1 g in 50 mL), and the desorption step was carried out with 1 M NaOH. This concentration was chosen on the basis of preliminary experiments and also previous studies [25,30]. After each adsorption/desorption step, the magnetite-based materials were dried overnight at 60 °C. The resulting dried material was then weighed and used for the next adsorption or desorption step.

### 2.5. Statistical Analysis

The regression analysis, along with the graphical representation of the experimental and calculated data, were performed using Excel 2016. The error bars in the displayed figures are the standard deviation of the triplicate experimental data.

## 3. Results and Discussion

### 3.1. Material Characterization

**Figure 2:**
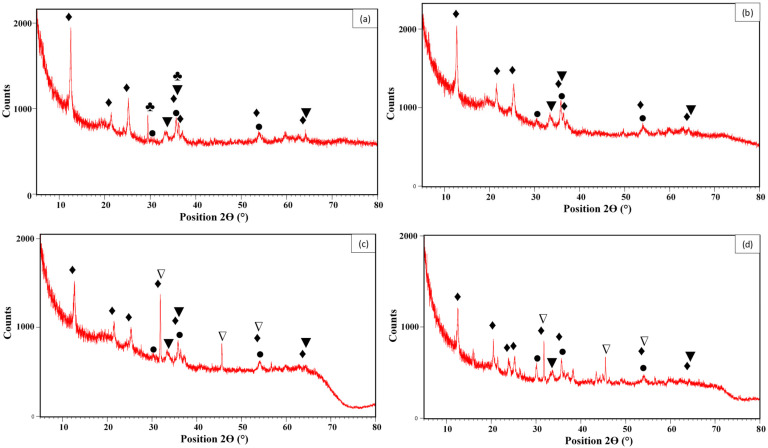
XRD spectra of the raw magnetite (**a**), MG-Fh (**b**), MG-Fh-La-5 (**c**), and MG-Fh-La-15. (**d**) (♦: Kaolinite; ●: magnetite; ▼: hematite; ♣: calcite; ∇: halite).

The XRD analyses (Figure 2a–d) show that the raw material was formed via a mixture of several crystalline phases including magnetite; hematite; kaolinite; and calcite, which were detected at 2θ of 30.4°, 36.3°, and 53.9°; 33.3°, 35.7°, and 64.2°; 12.5°, 21.4°, 25.2°, 35.7°, 36.3°, 53.9°, and 64.2°; and 29.5° and 36.3°, respectively (Figure 2a). Compared to the raw feedstock, the MG-Fh spectrum (Figure 2b) shows a relatively similar pattern, except for the disappearance of the two calcite peaks due to its dissolution by the modifying HCl acidic solution. The spectra related to the treated materials with lanthanum solutions at percentages of 5% (Figure 2c) and 15% (Figure 2d) are quite similar to that of the MG-Fh, but with the apparition of new peaks, detected at 2θ of 31.8°, 45.6°, and 54.1° corresponding to halite. The formation of this component is due to Na^+^ and Cl^-^ ions from the modification protocol (see Section 2.2). It is important to underline that no clear peaks related to La-based crystalline forms were observed in the spectra of MG-Fh-La-5 and MG-Fh-La-15, suggesting that they may be deposited on their surface but as amorphous phases. A similar observation was reported by Fu et al. [25] for a lanthanum-modified natural Chinese magnetite.

**Figure 3:**
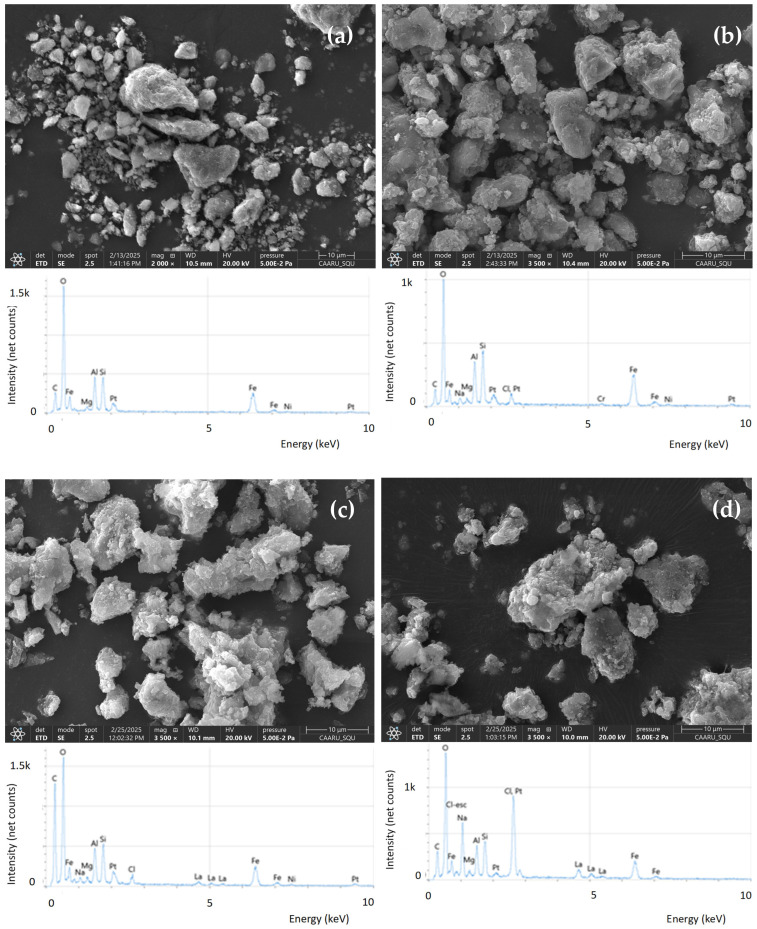
SEM/EDS analyses of raw magnetite (**a**), MG-Fh (**b**), MG-Fh-La-5 (**c**), and MG-Fh-La-15 (**d**).

The XRD results were confirmed via the XRF analyses (Table 1) that clearly show that the raw feedstock is not a pure magnetite. Indeed, besides iron, which has the highest content (12.4%), relatively important amounts of silicon (7.9%) and aluminum (6.5%) were measured in the feedstock (Table 1). These two latter elements are intrinsic components of kaolinite (Al_2_Si_2_O_5_(OH)_4_), whose presence was clearly identified in the XRD analysis of the feedstock (Figure 2). Therefore, based on XRD and XRF analyses, the feedstock is a mixture of magnetite, kaolinite, hematite, and other impurities [31]. Moderate contents of Mg, Cr, Ca, and Ni were measured in the feedstock material. Low contents of Zn (0.01%) and Cd (0.001%) exist in the raw material, while other heavy metals such as Pb and Hg were not detected (Table 1). Also, the P element was not measured in the raw feedstock. The magnetite transformation into MG-Fh significantly decreased the contents of all elements, except for O and Cl, whose contents increased from 69.1% to 75.4% and 0.02% to 0.43% (Table 1). This finding is due to the dissolution of these elements by the used acidic solution. Moreover, the use of HCl solution for the modification of the feedstock contributed to the increase in Cl contents in MG-Fh. The MG-Fh modification with lanthanum made this effect pronounced, where the O and Cl contents reached 84.9% and 1.95% for MG-Fh-La-15 (Table 1). This observation is confirmed by the SEM/EDS analyses (Figure 3). It shows that the raw feedstock is formed of large particles and is mainly formed with O, Fe, Si, Al, Mg, Ca, and other impurities (Figure 3a). The acidification process decreased the particles’ size without a clear impact on the material porosity and at the same time, a peak of Cl appeared due to the use of HCl reagent (Figure 3b). The La modification indicates the appearance of small and shiny particles on the surface of MG-Fh-La-5 (Figure 3c) and MG-Fh-La-15 (Figure 3d), corresponding most probably to lanthanum oxides. The success of La loading on the materials’ surface was confirmed by the EDS analyses, where new significant peaks of La were detected, especially for MG-Fh-La-15 (Figure 3c,d).

The acid treatment of the raw feedstock allows for the obtainment of a MG-Fh material with enhanced textural characteristics (Table 1). Indeed, the BET surface area and the total pore volume (TPV) values were evaluated to be 87.0 m^2^ g^−1^, and 0.084 cm^3^ g^−1^, respectively. They are around 96.3% and 86.6% higher than the values measured for the natural feedstock. A comparable BET surface area (94.7 m^2^ g^−1^) was reported for a Chinese magnetite decorated with Fh [25]. These properties’ enhancement confirms the natural feedstock coating with ferrihydrite nanoparticles, whose surface area was evaluated by Liu et al. [22] to be 219 m^2^ g^−1^, and by Mendez and Hiemstra [32] to be more than 600 m^2^ g^−1^. The modification of MG-Fh with lanthanum at a percentage of 5% slightly increased its BET surface area (reaching 88.8 m^2^ g^−1^), but significantly raised its total porosity volume by more than 69%, reaching a value of 0.142 cm^3^ g^−1^ (Table 1). This observation suggests the deposition of lanthanum oxides onto the MG-Fh surface and also the pores’ enlargement due to the calcination process at 200 °C. However, a further increase in the lanthanum percentage to 15%, on the contrary, led to decreases in the BET surface area and pore volume to 71.5 m^2^ g^−1^ and 0.103 cm^3^ g^−1^, respectively (Table 1). This is most likely due to the blocking of this material’s pores by the generated lanthanum oxides during the treatment process. Such behavior was observed by Fu et al. [25] and Xu et al. [33] after modification with lanthanum from a Chinese MG-Fh and a ferrate magnetic biochar from crab shells, respectively, and also by Yi et al. [34] for a sawdust-derived biochar co-modified with lanthanum and iron.

**Figure 4:**
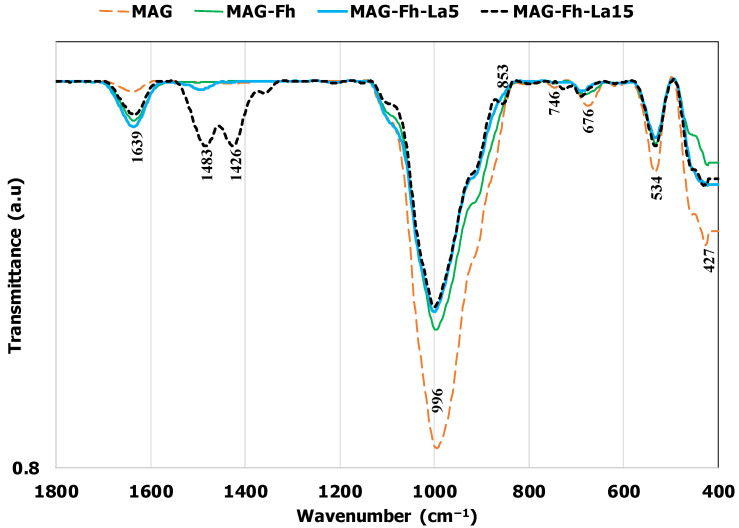
FTIR analyses of the magnetite-based materials.

Regarding the surface chemistry properties, it appears that the higher the La percentage, the lower the pHpzc of the La-modified materials (Table 1). These values were evaluated to be 7.14 and 6.31 for MG-Fh-La-5 and MG-Fh-La-15, respectively, indicating that for acidic effluents, these materials are mainly positively charged and will thus favor P recovery through electrostatic attraction [35]. A comparable value (6.35) was found for a La-modified magnetite from China [12]. Moreover, the FTIR analyses confirm that the raw feedstock is a complex material that involves several functional groups (Figure 4). They include Fe-O stretching vibrations at 427 cm^−1^ and 676 cm^−1^ [16,36,37], Al-O-Si and Si-O stretching bands from kaolinite at 534 cm^−1^ and 996 cm^−1^, respectively [38,39], a small Ca-O band from calcite at 746 cm^−1^ [40,41], and a hydroxyl stretching vibration (-OH) at 1639 cm^−1^ [15,16,37]. The MG-Fh has a similar spectrum to MAG, except for the disappearance of the Ca-O peak (at 746 cm^−1^) (Figure 4). This result is in agreement with the XRD analyses, which showed that the acid treatment of the MAG induced calcite dissolution (see Figure 2a,b). Moreover, three new peaks were detected after the MG-Fh modification with La(NO_3_)_3_, and especially for MG-Fh-La-15 (Figure 4). For instance, the peak detected at 853 cm^−1^ corresponds to a La-OH vibration [25,42]. Moreover, the peak observed at 1483 cm^−1^ can be attributed to the residual nitrates (NO_3_^−^) after the calcination of La(NO_3_)_3_x6H_2_O [43,44]. Finally, the peak at 1426 cm^−1^ indicates that La^3+^ ions may be incorporated into the structure of the La-modified MG-Fh [43]. This surface richness should contribute to the enhancement of P recovery from aqueous solutions [45].

### 3.2. Phosphorus Recovery Experimental Results

#### 3.2.1. Effect of Contact Time Kinetic Study

**Figure 5:**
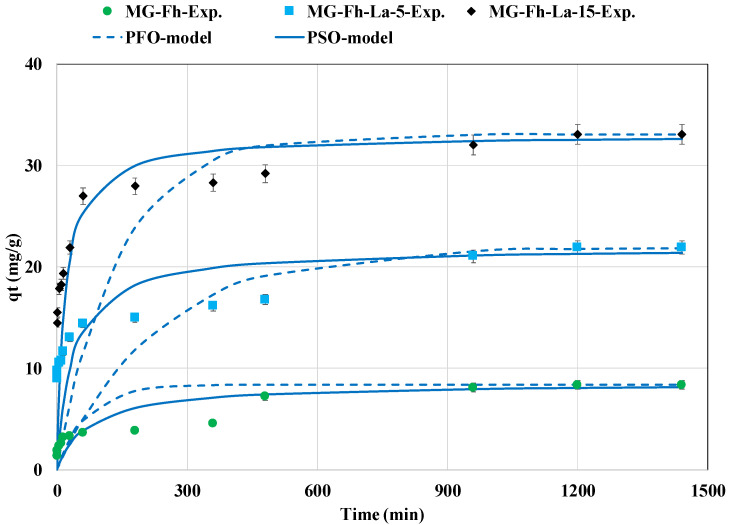
Phosphorus recovery kinetics of the three magnetite-based materials.

Figure 5 shows the P kinetic removal by MG-Fh, MG-Fh-La-5, and MG-Fh-La-15. It can be clearly seen that this process is dependent on the contact time. In fact, at the beginning (first hour), the recovered P rate greatly increased vs. time and reached around 43.7%, 65.9%, and 81.7% of the total recovered quantity for MG-Fh, MG-Fh-La-5, and MG-Fh-La-15, respectively (Figure 5). These high rates are ascribed to the important availability of sorption active sites and P molecules [46]. This phase corresponds to the P diffusion through the boundary layer surrounding the adsorbents’ particles. A second phase with lower adsorption rates is observed between 1 and 16 h. It corresponds to P intraparticle diffusion inside the mesopores and micropores of the adsorbents [47]. Finally, a quasi-equilibrium state is observed at contact times between 20 and 24 h, where the sorption active sites (at the surface of the tested adsorbents or inside the pores) are fully saturated. For all magnetite-based adsorbents, the boundary layer diffusion step is the limiting stage, since the related diffusion coefficients (D_f_) are smaller than those related to the intraparticle diffusion phase (D_ip_) (Table 2). Equivalent findings were reported for P recovery via Mg-modified date palm frond material [28].

The equilibrium time for the tested materials was assessed to be around 20 h. This duration is higher than those reported for a La-modified sheep dung activated carbon (2h) [42], a La-modified dewatered sludge-derived biochar (4 h) [43], and a La-modified MG-Fh from China (6 h) [25]. However, it is equivalent to that required for P recovery via a La-modified oak derived biochar [48] and a Mg-modified magnetite [17]. In our case, to reduce the energetic cost related to the agitation process, a contact time of only 6 h can be used. At this time, the recovered P amount represents more than 54%, 73%, and 85% of the total adsorbed quantity for MG-Fh, MG-Fh-La-5, and MG-Fh-La-15, respectively. It is important to underline that the amount of P recovered at equilibrium increases with the La percentage. In fact, the highest amount was evaluated to be 33.0 mg g^−1^ for MG-Fh-La-15, which is 1.5 and 3.9 times higher than that of MG-Fh-5 and MG-Fh, respectively. This result can be attributed to the enhancement of the materials’ properties with the increase in the La contents (See Section 3.1). This finding will be discussed in depth in the isotherm section (Section 3.2.5).

Table 2 depicts the kinetic models’ parameters for P recovery via the studied materials. It appears that for all materials, the PSO model fits the measured data better than the PFO model. In fact, the calculated MAPD and correlation coefficients between the experimental and predicted curves for the PSO model were, respectively, lower and higher than those for the PFO model (Table 2). For instance, for MG-Fh-La-15, the calculated MAPD and R^2^ by the PSO model were evaluated to be 26.7% and 0.941, while these parameters were assessed by the PFO model to be 48.1% and 0.909 (Table 2). In addition, for all materials, the calculated adsorbed amounts at equilibrium by the PSO model are close to the experimental ones, with gaps of only 2.5% for MG-Fh, 0.1% for MG-Fh-La-5, and 0.05% for MG-Fh-La-15, respectively. This result suggests that the P recovery process may mainly include chemical mechanisms [49]. Similar findings were also reported for P recovery via a magnetic lanthanum-carbonate-modified attapulgite [49], a dual La-Zr-modified magnetite [15], and a Mg-modified magnetite [17]. It is worth mentioning that the calculated MAPD values were relatively high (between 47.6% and 51.1% for the PFO model and 26.7% and 41.4% for the PSO model), which is an indication of poor agreement between the measured and calculated adsorbed masses. This behavior is mainly due to (Figure 5) (i) the exceptionally high adsorption rate at the beginning of the assays (for times lower than 15 min) compared to the rest of the assays and (ii) the presence of a first quasi plateau (at times between 3 and 8 h), followed by a second one starting from 20 h.

#### 3.2.2. Effect of Initial Aqueous pH

**Figure 6:**
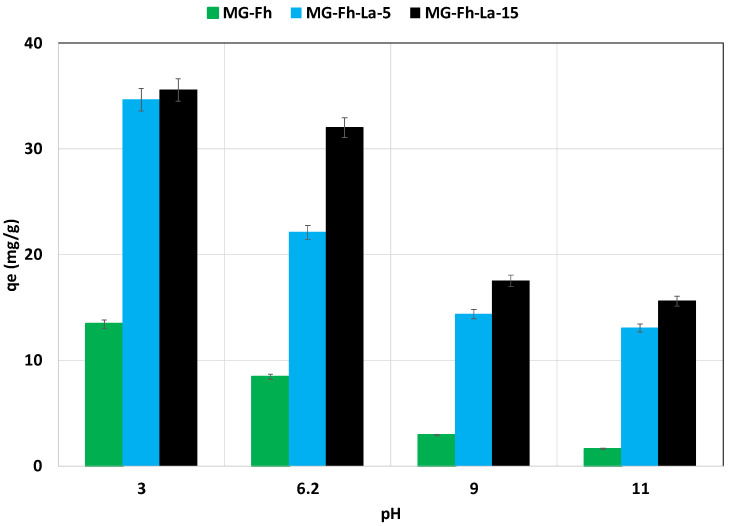
Initial pH effect on P recovery efficiency with the studied magnetite-based materials.

The effect of the initial pH on P’s recovery performance via the three materials was carried out under the experimental conditions given in Section 2.4.2. The results (Figure 6) show that for all materials, the amounts of P recovered decrease with the increase in the initial pH values. Indeed, raising the pH from 3 (highly acidic medium) to 11 (highly alkaline medium) decreased the recovered P amount by 87.4%, 62.3%, and 56.2% for MG-Fh, MG-Fh-La-5, and MG-Fh-La-15, respectively (Figure 6). This finding may be mainly imputed to the fact that the studied materials became negatively charged for pH values higher than their pHpzc, which are in the range of 6.31–7.14 (see Table 1), and will consequently repulse the negatively charged P ions. The possible forms of phosphate ions are H_3_PO_4_, H_2_PO_4_^−^, HPO_4_^2−^ and PO_4_^3−^, and the corresponding pKa1, pKa2, and pKa3 are equal to 2.12, 7.20, and 12.36, respectively [50]. Moreover, increasing the pH values results in a net increase in hydroxyl ions in the solution, which can lead to an adsorption competition with P anions for the active adsorption sites [47]. A similar behavior was reported for P recovery via a La-modified magnetite decorated with ferrihydrite [25], a La-modified magnetite [12,14], and a Mg/Fe-coated magnetite [18].

#### 3.2.3. Effect of Adsorbent Doses

**Figure 7:**
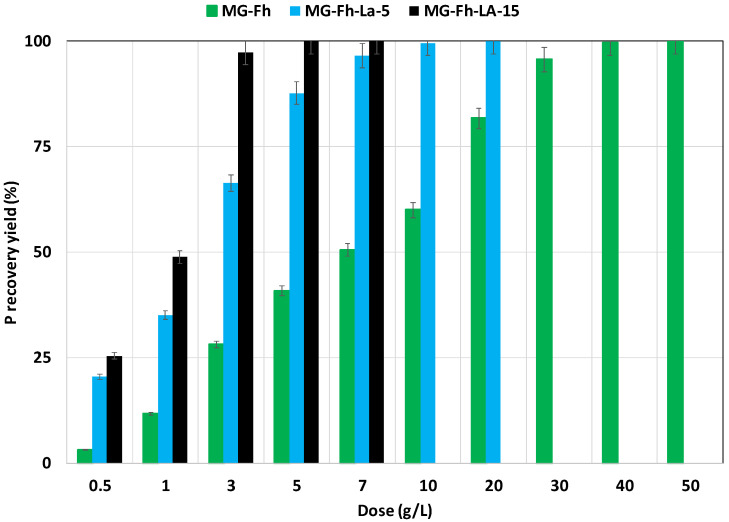
Effect of the magnetite-based material doses on P recovery performance from aqueous solutions.

The impact of the materials’ doses on P’s recovery performance was assessed at an initial concentration of 66 mg L^−1^, a contact time of 24 h, and a natural pH (without adjustment). Figure 7 clearly shows that the P recovery yields increase with the rise in the used doses. Indeed, the lowest dose (0.5 g L^−1^) permits P recovery yields of only 3.1%, 20.5%, and 25.4% for MG-Fh, MG-Fh-La-5, and MG-Fh-La-15, respectively. Given the fact that the P recovery efficiency increase is favored by the increase in the percentage of La used, the required dose to achieve full recovery of the contained P in the aqueous solution was assessed to be only 5 g L^−1^ for MG-Fh-La-15. This dose increases to 10 g L^−1^ for MG-Fh-La-5 and reaches 40 g L^−1^ for the less efficient material (MG-Fh) (Figure 7). This result is expected because increasing the adsorbent dose results in the presence of more active adsorption sites, which will increase the exchange probability with the P ions. Comparable trends were observed for P recovery via numerous La-modified materials such as a mixture of magnetite and attapulgite [49], and a peanut-shell-derived biochar [51].

#### 3.2.4. Effect of the Presence of Anions

**Figure 8:**
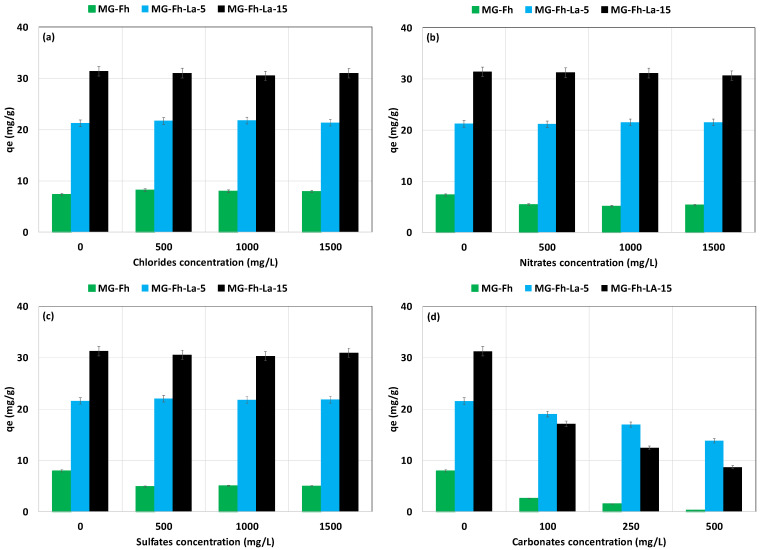
Effect of the presence of chlorides (**a**), nitrates (**b**), sulfates (**c**), and carbonates (**d**) on P recovery efficacy via the four magnetite-based materials.

The impact of the presence of competitive ions (Cl^−^, NO_3_^−^, SO_4_^2−^, and CO_3_^2−^) at various initial concentrations on P recovery was assessed at an initial P concentration of 66 mg L^−1^, a contact time of 24 h, and a natural pH (Figure 8). It can be clearly seen that for all tested materials, no important effect was observed for Cl^−^, NO_3_^−^, or SO_4_^2−^, where the amount of recovered P remained almost constant. This is due to the high electron pair donor ability of P molecules compared to Cl^−^, NO_3_^−^, and SO_4_^2−^ anions [52]. This highlights the strong selectivity of our materials towards P, and also suggests its feasibility for real wastewater treatment. Similarly, for these anions, no significant effect was previously observed for P recovery via a La-modified magnetite [12], a La-magnetic nanocomposite of sugarcane bagasse cellulose [53], or a magnetite/lanthanum carbonate co-modified activated attapulgite composite [49].

Contrarily, the presence of CO_3_^2−^, even at relatively low concentrations (100 mg L^−1^), significantly decreased the materials’ efficiency in recovering P from the aqueous solutions. The highest decrease was observed for the largest tested CO_3_^2−^ concentration (500 mg L^−1^) (Figure 8). For instance, at a CO_3_^2−^ concentration of 500 mg L^−1^, the decrease in P recovery was evaluated to be around 96.8%, 35.8%, and 72.2%, for MG-Fh, MG-Fh-La-5, and MG-Fh-La-15, respectively. This finding may be due to the possible combination of La^3+^ with CO_3_^2−^, which partially forms a solid precipitate (La_2_(CO_3_)_3_) instead of La(OH)_3_, resulting in a net decrease in the P recovered by the latter component [54]. A similar trend was also found for P recovery via numerous engineered materials [17,49,51,54].

#### 3.2.5. Effect of Initial Concentration Isotherm Study

**Figure 9:**
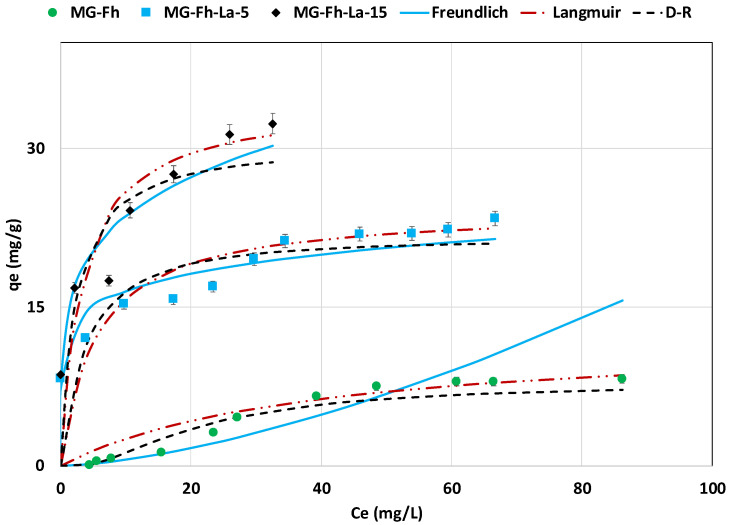
Isotherm observed and predicted data with Langmuir, Freundlich, and D-R models of P recovery with the magnetite-based materials.

The impact of P’s initial concentration on its recovery via the three magnetite-based adsorbents was carried out for a non-adjusted pH, a dose of 1 g L^−1^, and a contact time of 24 h. The experimental results (Figure 9) indicate that the amount of recovered P increased with the increase in the P aqueous concentration. Indeed, the largest amounts of recovered P were measured for the highest initial P concentrations and evaluated to be 8.2, 23.4, and 32.3, mg g^−1^ for MG-Fh, MG-Fh-La-5, and MG-Fh-La-15, respectively (Figure 9). This observation can be imputed to the higher concentration gradients between the aqueous solutions and the adsorbents, which result in greater diffusion fluxes into the materials’ porosities [41]. The observed goodness-of-fit of the isotherm with the Langmuir, Freundlich, and D-R models can be deduced from Figure 9 and Table 3.

It can be concluded that for the three materials, the Freundlich model fits better with the experimental data, since it exhibits the highest correlation coefficients (R^2^) and the lowest MAPD (Table 3). For instance, for the most efficient tested material (MG-Fh-La-15), the calculated R^2^ and MAPD by the Freundlich model were equal to 0.934 and 7.5%, respectively. These parameters were evaluated to be 0.866 and 23.8% for the Langmuir model and 0.827 and 22.7% for the D-R model (Table 3). This indicates that the P recovery adsorption onto the La-modified magnetite occurs on multilayers and heterogeneously on the materials’ surface [55]. Moreover, for these three materials, the P adsorption process is favorable, since both their calculated Freundlich constant “n” values are much higher than 1 (Table 3), and their Langmuir constants ($R_{L}=\frac{1}{1+K_{L*C_{0}}}$) are lower than 1.

For the tested materials, the D-R’s free adsorption energy (E) calculated values increase with the rise in the La percentage from 1.788 kJ mol^−1^ for MG-Fh to 5.687 kJ mol^−1^ for MG-Fh-La-15 (Table 3). However, these values remain lower than 8.0 kJ mol^−1^ (Table 3), suggesting that the P recovery process via these adsorbents also involves physical processes [56]. This may be due to the relatively low La percentages used (up to 15%). Adsorption energy values in this same range were found for studies on P recovery with a graphene oxide modified with magnetic nanoparticles [57], and with an aluminum–titanium bimetal oxide composite [58]. However, other studies have reported “E” values between 8.0 and 16.0 kJ mol^−1^, indicating the involvement of chemical processes. This was the case for P recovery via a mixture of magnetite and zeolite modified with a high concentration of zirconium dichloride oxide (ZrOCl_2_x8H_2_O) [56].

The Langmuir model’s adsorption capacities onto the MG-Fh, MG-Fh-La-5, and MG-Fh-La-15 are evaluated to be 12.4, 24.3, and 34.5 mg g^−1^, respectively (Table 3). The increase in the P recovery capacity with the rise in the La percentage is mainly due to the net improvement of the related physico-chemical properties. For example, compared to the raw feedstock (MAG), the BET surface area and total pore volume increased by 61.4% and 128.9%, respectively, when the La treatment percentage was fixed to 15% (see Table 1). Moreover, the surface functionality of the La-modified materials was greatly improved, especially for MG-Fh-La-15 with the apparition of new FTIR peaks corresponding to La–OH vibration, and incorporated La^3+^ inside its structure (see Section 3.1).

It is worth mentioning that the three materials’ adsorption capacities calculated using the D-R model are lower than those estimated using Langmuir’s model (Table 3). This may be attributed to the stringent assumptions of this model, especially regarding the assumption of the homogenous microporous structure of the adsorbents [55,59].

To liken the P recovery efficiency values of our adsorbents with those reported in the scientific literature, a comparison based on the Langmuir model’s adsorption capacity was performed (Table 4). It can be deduced that the MG-Fh-La-15 is considered a promising material for P recovery from aqueous solutions. Indeed, its adsorption capacity is 57.5, 10.1, 2.0, and 1.4 times higher than raw magnetite [60], a raw magnetite-coated biochar [61], a La-modified synthetic magnetite [62], and a La-modified synthetic magnetite at a percentage of 20% [14]. It is lower than that of modified magnetite with much higher La percentages [12] (Table 4).

The effect of aqueous solution temperature on the P recovery efficiency using our La-modified materials was not evaluated in the current study. However, according to previous studies, it seems that increasing this temperature usually results in an increase in the amount of P recovered using magnetite-based materials [14,65].

#### 3.2.6. Regeneration Study

**Figure 10:**
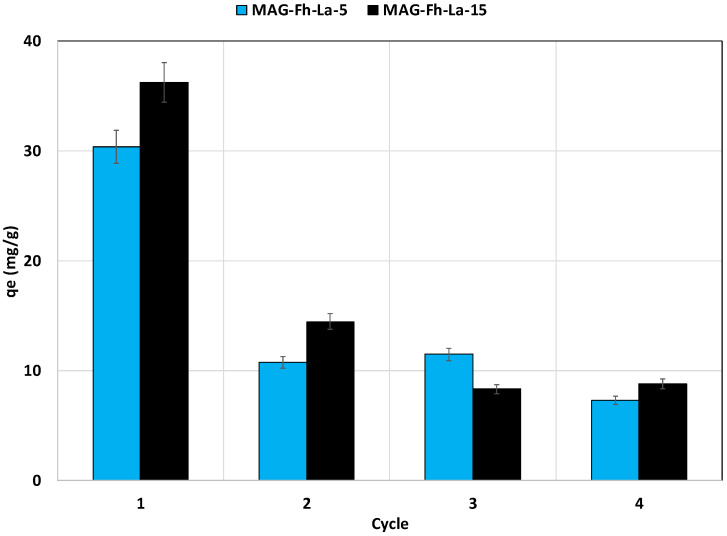
Regeneration capacity of the magnetite-based adsorbents using 1 M NaOH solution.

The regeneration study of the magnetite-based materials was performed as indicated in Section 2.4.4. The results (Figure 10) show that the regeneration capacity of all La-modified materials was relatively low. Indeed, in comparison with the first cycle, the P recovery efficiency significantly decreased in the second cycle by around 64.5% and 60.0% for MG-Fh-La-5, and MG-Fh-La-15, respectively. In the fourth cycle, the percentage decreases reached more than 76% and 75%, respectively. These high decreases in P recovery efficiency with the application of more adsorption/desorption cycles may be due to the fact that the P was mainly adsorbed through chemical mechanisms [66]. A relatively high P decrease percentage (55%) was reported for a La-modified natural magnetite [12].

#### 3.2.7. Effect of Using Real P-Doped Wastewater

**Figure 11:**
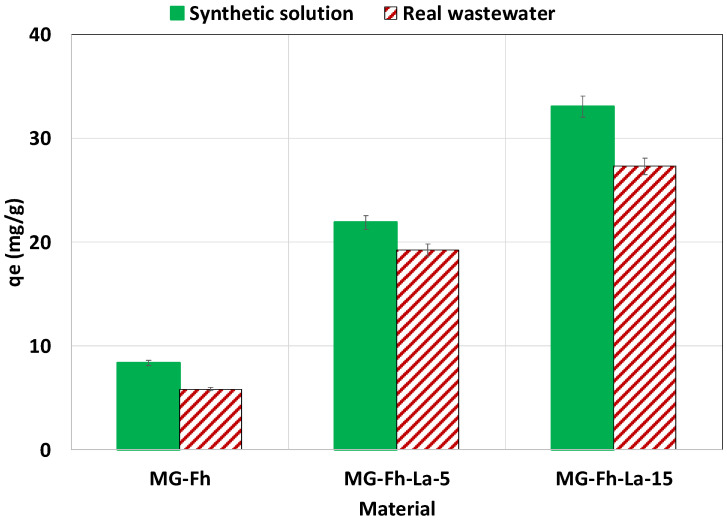
P recovery efficiency from actual wastewater in comparison with synthetic solutions.

The used wastewater is of a relatively good quality that satisfies the discharge norm (Table S2). Its initial P concentration was 4.1 mg L^−1^. This concentration was adjusted to 66 mg L^−1^ and then compared to the synthetic solution. The results (Figure 11) show that using wastewater instead of the synthetic solution has decreased the recovered amount of P by 43.2%, 13.9%, and 21.1% for MG-Fh, MG-Fh-La-5, and MG-Fh-La-15, respectively. This finding may be attributed the presence of competitive anions (i.e., CO_3_^2−^) and also dissolved organic matter [49,60]. However, the P-adsorbed amounts, especially by MG-Fh-La-15 (27.3 mg g^−1^), remain relatively high compared to various materials (see Table 4), and confirm its attractiveness for potential use in actual wastewater treatment. Few studies have investigated the efficiency of La-modified magnetite-based materials in recovering P from real media (i.e., wastewater or lake water). In this context, Fang et al. [14] showed that compared to synthetic solutions, the use of La(OH)_3_-modified magnetite reduces the amount of P adsorbed from a lake water sample by 34.7–58.3%. A comparable trend was observed for P recovery from a real secondary effluent (Beijing, China) via magnetite mineral microparticles [60]. However, these authors proved that this observed P recovery effectiveness reduction can be compensated for by using larger adsorbent amounts. It is important to underline that as observed when using synthetic solutions, the P desorption potential of the P-loaded materials from wastewater should be relatively low. Indeed, preliminary experiments indicated that this process is manly dependent on the NaOH concentration used.

### 3.3. P Recovery Mechanisms Exploration

**Figure 12:**
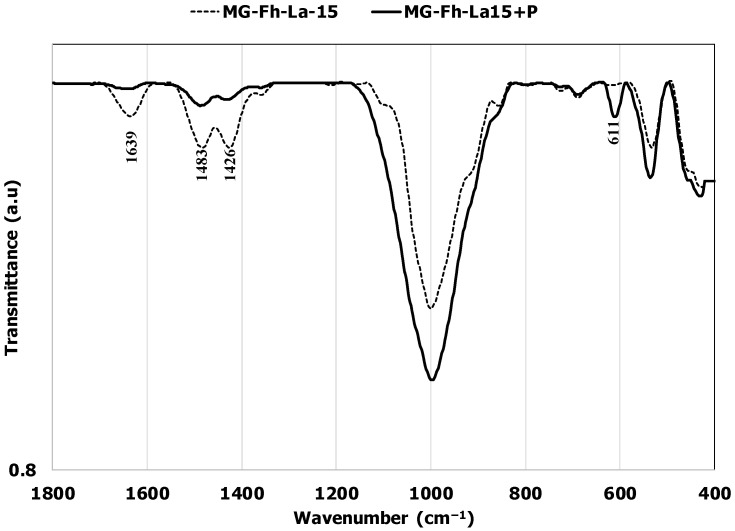
FTIR spectra of MG-Fh-La-15 before and after P adsorption.

The kinetic and isotherm modeling studies showed that P recovery using the La-modified materials involves both physical and chemical processes (see Section 3.2.1 and Section 3.2.5). Moreover, the investigation on the effect of pH has clearly shown that the P recovery process includes electrostatic interactions (see Section 3.2.2). This latter mechanism was also found to be involved in P recovery via La-modified magnetite [26,62] and other La-modified materials such as magnetic biochar [67]. The FTIR analysis of the La-modified materials showed that the complexation mechanism is also well involved in this process. Indeed, for MG-Fh-La-15, after P recovery, the intensity of the –OH peak at 1639 cm^−1^ and La^3+^ at 1426 cm^−1^ significantly decreased, indicating that they contributed to P recovery through complexation (Figure 12). At the same time, a new peak at 611 cm^−1^ appeared. It corresponds to the P–O–P bending vibration [43,68]. These results confirm P adsorption through complexation via our La-modified materials. The complexation mechanism was also reported to be involved in P recovery via a La-modified magnetite [25] and a magnetite/lanthanum carbonate co-modified activated attapulgite composite [49]. It is worth mentioning that the XRD analyses prove that no La–P crystalline phases were formed under the experimental conditions range used. This may be imputed to the relatively low La contents used (up to 15%). It is worth mentioning that Fu et al. [25] showed that X-ray photoelectron spectrometry (XPS) analyses of La-modified natural magnetite before and after P recovery confirm the formation La-O-P and Fe-O-P bonds. A similar finding was also reported for magnetite/lanthanum carbonate co-modified activated attapulgite composite [49].

## 4. Conclusions

The current research shows that natural feedstock from magnetite deposits (mixture of magnetite and kaolinite) may be turned into high value-added products. Indeed, this feedstock decoration with ferrihydrite followed by modification with lanthanum allowed for obtaining materials with enhanced structural, textural, and surface chemistry properties. These materials with high surface areas and functional-rich surfaces efficiently recover phosphorus from effluents under a wide range of experimental static conditions. The P adsorption capacity was favored in acidic media and significantly increased with the increase in the lanthanum percentage. The highest P recovery capacity (34.5 mg g^−1^) was found for a lanthanum percentage of 15%, which is much higher than various La-modified materials. This latter material conserves high P recovery efficiency, even from actual wastewater (27.3 mg g^−1^). P recovery seems to involve both physical and chemical mechanisms, such as electrostatic interactions and complexation with hydroxyl groups. These promising results should be confirmed through dynamic assays using either laboratory columns or continuous stirring reactors (CSTRs).

## Figures and Tables

**Table 1:** Main properties of the raw magnetite and its derived materials (SA: Brunauer–Emmett–Teller surface area; TPV: total pore volume; and ND: not detected).

Material	Mineral Contents (%)															pHpzc	BET Analysis	
	O	Fe	Si	Al	Mg	Cr	Ca	Ni	Mn	Cl	Zn	Cd	P	Pb	Hg		SA (m^2^ g^−1^)	TPV (cm^3^ g^−1^)
MAG	69.10	12.40	7.91	6.50	1.25	1.09	0.93	0.30	0.04	0.02	0.01	0.001	ND	ND	ND	-	44.3	0.045
MG-Fh	75.40	10.20	6.22	5.14	ND	0.94	0.20	0.24	0.03	1.43	0.01	0.001	ND	ND	ND	7.02	87.0	0.084
MG-Fh-La-5	80.50	6.72	4.96	4.11	ND	0.93	0.16	0.16	0.07	2.00	0.004	0.001	ND	ND	ND	7.14	88.8	0.142
MG-Fh-La-15	84.90	4.13	3.81	3.21	ND	0.97	0.12	0.11	0.07	1.95	0.002	<0.001	ND	ND	ND	6.31	71.5	0.103

**Table 2:** Kinetic models’ parameters for phosphorus recovery via the three magnetite-based materials (q_e,exp_ and q_e,pred_ are the experimental and predicted P adsorbed amounts at equilibrium, respectively).

	Parameter	MG-Fh	MG-Fh-La-5	MG-Fh-La-15
	q_e,exp_ (mg g^−1^)	8.36	21.89	33.05
PFO model	k_1_ (min^−1^)	0.0144	0.0043	0.0071
	R^2^	0.749	0.905	0.909
	MAPD (%)	47.6	51.1	48.1
PSO model	k_2_ (g mg^−1^ min^−1^)	0.0016	0.0012	0.0016
	q_e,pred_ (mg g ^−1^)	8.57	21.91	33.0
	R^2^	0.840	0.854	0.941
	MAPD (%)	41.4	36.2	26.7
Diffusion model	D_f_ (×10^−13^ m^2^ s^−1^)	0.80	0.68	1.14
	R^2^	0.883	0.983	0.982
	D_ip_ (×10^−13^ m^2^ s^−1^)	2.34	1.67	1.34
	R^2^	0.928	0.903	0.978

**Table 3:** Isotherm model parameters for phosphorus recovery via the magnetite-based materials.

Isotherm	Parameter	MG-Fh	MG-Fh-La-5	MG-Fh-La-15
Freundlich	n	0.66	7.22	4.72
	K_F_	0.017	11.993	14.464
	R^2^	0.777	0.902	0.934
	MAPE (%)	57.3	8.6	7.5
Langmuir	K_L_ (L mg^−1^)	0.026	0.184	0.297
	q_m,L,calc_ (mg g^−1^)	12.4	24.3	34.5
	R^2^	0.964	0.874	0.866
	MAPD (%)	331.4	14.8	23.8
D-R	q_m,D-R,calc_ (mg g^−1^)	7.8	21.4	29.9
	E (kJ mol^−1^)	1.788	4.729	5.687
	R^2^	0.960	0.806	0.827
	MAPD (%)	43.6	16.4	22.7

**Table 4:** Comparison of P recovery capacity via our magnetite-based materials with other engineered materials (C0: initial P concentration; D: adsorbent dose; t: contact time; T: temperature; and q_m,L_: Langmuir’s adsorption capacity: -: not given).

Adsorbent	Adsorption Experimental Conditions	q_m,L_ (mg g^−1^)	Reference
Magnetite mineral microparticles (average particle size: 34 μm)	C_0_ = 0.5–9 mg L^−1^; pH = 7.0; D = 2 g L^−1^; t = 24 h; T = 25 °C	0.6	[60]
La-modified commercial resin at a percentage of 6%	C_0_ = 155 mg L^−1^; pH= 7.0; D = - g L^−1^; t = - h; T = RT °C	1.3	[63]
Synthetic magnetite-coated commercial biochar	C_0_ = 25–500 mg L^−1^; pH = 6.5; D = 10 g L^−1^; t = 24 h; T = -	3.4	[61]
La-modified natural vesuvianite at a percentage of 14%, China	C_0_ = 1–5 mg L^−1^; pH = 7.1; D = 0.3 g L^−1^; t = 40 h; T = 20 °C	6.7	[64]
La-modified synthetic magnetite at a percentage of 5%	C_0_ = 1–10 mg L^−1^; pH = 7.0; D = 0.1 g L^−1^; t = 2 h; T = 25 °C	13.4	[14]
La-modified synthetic magnetite at a percentage of 10%		12.3	
La-modified synthetic magnetite at a percentage of 20%		25.4	
La-modified synthetic magnetite	C_0_ = 5–30 mg L^−1^; pH = 7.0; D = 0.4 g L^−1^; t = 24 h; T = 25 °C	17.3	[62]
La-modified natural magnetite decorated with ferrihydrite at a percentage of 20%, China	C_0_ = 2–120 mg L^−1^; pH = 6.28; D = 1 g L^−1^; t = 24 h; T = 25 °C	44.8	[25]
La/Zr-modified synthetic magnetite at La:MG and Zr:MG percentages of 64% and 40%, respectively	C_0_ = 2.5–50 mg L^−1^; pH = 2.0; D = 0.25 g L^−1^; t = 24 h; T = 25 °C	49.1	[15]
La-modified synthetic magnetite and attapulgite at a percentage of 28%	C_0_ = 1–300 mg L^−1^; pH = 7.0; D = 1 g L^−1^; t = 24 h; T = 25 °C	51.7	[49]
La-modified synthetic magnetite at a percentage of 128%	C_0_ = 0.5–250 mg L^−1^; pH = 7.0; D = 0.1 g L^−1^; t = 5 h; T = 23 °C	253.8	[12]
La-modified natural magnetite decorated with ferrihydrite at a percentage of 0%, Oman	C_0_ = 5–100 mg L^−1^; pH = natural; D = 1 g L^−1^; t = 24 h; T = RT	12.4	This study
La-modified natural magnetite decorated with ferrihydrite at a percentage of 5%, Oman		24.3	
La-modified natural magnetite decorated with ferrihydrite at a percentage of 15%, Oman		34.5	

## Data Availability

The original contributions presented in this study are included in the article/Supplementary Material. Further inquiries can be directed to the corresponding author.

## Supplementary Materials

The following supporting information can be downloaded at: https://www.mdpi.com/article/10.3390/ma18102283/s1. Table S1: Kinetic and isotherm model equations used for the fitting of experimental data, Table S2: Main properties of the used real wastewater.
