# Supplementary material for: Synthesis of Lanthanum-Modified Natural Magnetite: Characterization and Valorization for Phosphorus Recovery from Aqueous Solutions
Source: Materials (Basel). 2025 May 14;18(10):2283. doi: 10.3390/ma18102283 (PMC12113161; doi:10.3390/ma18102283)
Supplement: Supplementary file 1 [file materials-18-02283-s001.zip › materials-3616251-supplementary.pdf]

Supplementary Materials

Synthesis of Lanthanum-Modified Natural Magnetite: Characterization and Valorization for Phosphorus Recovery from Aqueous Solutions

Hamed Al-Nadabi [1], Salah Jellali [1,*], Wissem Hamdi [2], Afrah Al-Tamimi [1], Ahmed Al-Raeesi [1], Ahmed Al-Sidairi [1], Waleed Al-Busaidi [3], Ahlam Al-Hanai [1], Khalifa Al-Zeidi [1], Malik Al-Wardy [1] and Mejdi Jeguirim [4]

[1] Centre for Environmental Studies and Research, Sultan Qaboos University, Al-Khoud 123, Muscat P.O. Box 17, Oman; hamed@squ.edu.om (H.A.-N.); achemtamimi@gmail.com (A.A.-T.); aalraeesi@squ.edu.om (A.A.-R.); sidairi@squ.edu.om (A.A.-S.); aalhinai@squ.edu.om (A.A.-H.); alzeidi@squ.edu.om (K.A.-Z.); mwardy@squ.edu.om (M.A.-W.)

[2] Higher Institute of the Sciences and Techniques of Waters, University of Gabes, Gabes 6033, Tunisia; wissemhemdi@yahoo.fr

[3] College of Agricultural and Marine Sciences, Sultan Qaboos University, Al-Khoud 123, Muscat P.O. Box 17, Oman; waleedm@squ.edu.om

[4] The Institute of Materials Science of Mulhouse (IS2M), UMR 7361, University of Haute Alsace, CNRS, P.O. Box 2488, 68100 Mulhouse, France; mejdi.jeguirim@uha.fr

* Correspondence: s.jelali@squ.edu.om

Table S1. Kinetic and isotherm model equations used for the fitting of experimental data (q_t: adsorbed amount at time t; q_e: adsorbed amount at equilibrium; k_1: kinetic recovery rate of the pseudo-first-order model, k_2: kinetic recovery rate of the second-order model, $q_{e,calc}$: calculated adsorbed amount at equilibrium; D_f: film diffusion coefficient; D_{ip}: intraparticle diffusion coefficient; a: adsorbent's average particle size; $q_{m,L}$: Langmuir's adsorption capacity; C_e: equilibrium concentration in water; K_L: Langmuir's coefficient; K_F: Freundlich coefficient; n: Freundlich parameter; $q_{m,D\text{-}R}$: adsorption capacity of D-R model; β: constant related to adsorption energy; ε: Polanyi potential. .

Name	Equation
Kinetic model	
Pseudo first-order model (PFO)	$\frac{dq_t}{dt} = k_1(q_e - q_t)$
Pseudo second-order (PSO)	$\frac{t}{q_t} = \frac{t}{k_2\, q_{e,calc}{}^2} + \frac{t}{q_{e,calc}}$
Boundary layer diffusion	$\frac{q_t}{q_e} = 6\,(\frac{D_f}{\pi a^2})^{1/2}\,\sqrt{t}$
Intraparticle diffusion	$Ln\left(1 - \frac{q_t}{q_e}\right) = Ln\left(\frac{6}{\pi^2}\right) - \left(\frac{D_{ip}\,\pi^2}{a^2}\right) x\, t$
Isotherm model	
Langmuir	$q_e = \frac{q_{m,L}\;K_L\;C_e}{1 + K_L\;C_e}$
Freundlich	$q_e = K_F\, C_e{}^{1/n}$
Dubinin-Radushkevich (D-R)	$q_e = q_{m,D-R}\exp[-\beta\;\varepsilon^2]$

Table S2. Main properties of the used actual wastewater.

Parameter	Value
pH (-)	8.3
Electrical conductivity (mS cm^{-1})	2.2
Suspended solids (mg L^{-1})	3.9
Total dissolved salts (mg L^{-1})	1,043
Chlorides (mg L^{-1})	380.0
Nitrates (mg L^{-1})	6.0
Sulfates	146.0
P-PO_4 (mg L^{-1})	4.1
Calcium (mg L^{-1})	35.9
Sodium (mg L^{-1})	285.5
Potassium (mg L^{-1})	24.9
Magnesium (mg L^{-1})	41.3
